# High CTC-TRPC5 Expression Significantly Associated With Poor Prognosis in Radical Resected Colorectal Cancer Patients

**DOI:** 10.3389/fmolb.2021.727864

**Published:** 2021-08-04

**Authors:** Dongyan Cai, Na Li, Linfang Jin, Xiaowei Qi, Dong Hua, Teng Wang

**Affiliations:** ^1^ Department of Oncology, Affiliated Hospital of Jiangnan University, Wuxi, China; ^2^ Wuxi Medical College, Jiangnan University, Wuxi, China; ^3^ Department of Pathology, Affiliated Hospital of Jiangnan University, Wuxi, China; ^4^ Department of Oncology, Wuxi People’s Hospital Affiliated to Nanjing Medical University, Wuxi, China

**Keywords:** colorectal cancer, TRPC5, CTC, DFS, prognosis

## Abstract

Recurrence is the main reason of treatment failure of redical resected colorectal cancer (CRC). Although some factors including staging and differentiation have been proven to useful for recurrence evaluation, prognosis of certain patients does not conform to this evaluation approach. Circulating tumor cells (CTC) have been found to have prognostic value in CRC, and previous studies on CTC have primarily focused on their numbers. CTC are functionally heterogeneous cell populations, and different CTC subgroups may have different functions and clinical values. In our previous study, we discovered that elevated expression of the transient receptor potential channel TRPC5 was associated with a significantly poor prognosis in CRC. In this study, we collected peripheral blood from CTC-positive CRC patients, identified the TRPC5 protein expression on CTC (CTC-TRPC5), and analyzed the relationship between CTC-TRPC5 expression levels and the prognosis. The results showed that CTC-TRPC5 level is significantly related to the T stage and differentiation of tumors. High level of CTC-TRPC5 is more common in a high T stage as well as poorly differentiated tumors and is significantly associated with shorter disease free survival (DFS). The median DFS of CRC patients with high and low CTC-TRPC5 level was 17.1 and 22.0 months, respectively (*p* < 0.05). This study revealed a clinically significant CTC subgroup of CRC, providing a new indicator for clinical evaluation of CRC prognosis.

## Introduction

Colorectal cancer (CRC) is currently one of the most common gastrointestinal malignancies in the world, with its morbidity and mortality rate ranking third and second among all malignant tumors (Sung et al., 2021). Treatment for non-metastatic CRC involves a comprehensive strategy with surgery as the mainstay. After surgery, some patients will experience recurrence and metastasis, which will lead to cancer-related deaths in most cases. There are currently a variety of clinical and pathological factors including staging, differentiation which have been proven to be related to recurrence risks (Benson et al., 2021). However, current commonly used clinical indicators are unable to perfectly distinguish CRC patients with different recurrence risks.

Circulating tumor cells (CTC) are tumor cells that are separated from solid tumor lesions which enter the blood circulatory system, where they can be detected in peripheral blood (Smit et al., 2021). There are currently various technologies available that can separate CTC from blood using biophysical methods (deformability, size, density, and surface charge) and/or immunoaffinity status (Khetani et al., 2018;Habli et al., 2020;Yang et al., 2021). Previous studies have found that the number of CTC in the peripheral blood of patients with CRC after surgery is significantly related to the prognosis (Rahbari et al., 2010;Iinuma et al., 2011;Bork et al., 2015). CTC is also heterogeneous due to the recognized heterogeneity of CTC-derived tumors (Keller and Pantel, 2019; Tellez-Gabriel et al., 2019; Menyailo et al., 2020). However little is known about the heterogeneity of CTC in CRC.

Transient receptor channel protein C5 (TRPC5) is a calcium ion channel expressed in the cell membrane which affects the biological functions of cells through the regulation of calcium ion influx (Ma et al., 2012). We previously discovered that high expression of TRPC5 in CRC cells was related to drug resistance (Wang et al., 2015), while high expression of TRPC5 in CRC tissues was related to a worse prognosis (Chen et al., 2017). The study aimed to investigate TRPC5 protein expression on CTC (CTC-TRPC5) of CRC, whether there is a difference in expression, and whether this difference has clinical significance.

## Materials and Methods

### Patients and Follow up

Patients who underwent radical CRC surgery in Affiliated Hospital of Jiangnan University between October 2018 and April 2020 were selected. Three weeks after the operation, 7 ml of peripheral blood was drawn, then CTC was detected and isolated. Criteria included: 1) did not receive neoadjuvant chemoradiation and/or other immunotherapy/targeted therapy before surgery, 2) did not receive adjuvant chemoradiation and/or other immunotherapy/targeted therapy before CTC detection after surgery, 3) sufficient surgical tissue specimens can be obtained for immunohistochemical detection, 4) at least one CTC can be detected in the peripheral blood, and 5) with complete follow up of at least 6 months or recurrence. Clinical and pathological data of all patients, including age, gender, pathological stage, tumor differentiation, and tumor location were collected. After the operation, regular follow-ups, including colonoscopy as well as chest, abdomen, and pelvic CT examinations were performed. Recurrence is defined as anastomotic confirmed through gastroscope and/or regional lymph node/distant metastasis confirmed through imaging examination. The last follow-up was in June 2021. Ethical permission was obtained from the Ethics Committee of the Affiliated Hospital of Jiangnan University and conformed to the provisions of the Declaration of Helsinki (as revised in Fortaleza, Brazil, October 2013).

### CTC Detection by a Size-Based Platform

CTC were isolated using the ISET technology (Vona et al., 2000). Firstly, 5 ml of peripheral blood samples were collected and diluted in a 3 ml buffer containing 0.2% paraformaldehyde. After that, 200 μL phosphate-buffered saline (PBS) containing 8% formaldehyde (Leagene, Beijing, China) was added to resuspend the samples, and the samples were fixed for 10 min. Then, 8 ml of the diluted blood was filtered through a membrane with 8 μm diameter circular pores. The enriched CTC that were obtained were used for TRPC5 protein examination.

### CTC-TRPC5 Examination

The primary antibody anti-TRPC5 (1:100) (PA5-77310, Invitrogen, Camarillo, CA, United States) and the secondary antibody (1:1000) (Alexa Fluor 568, A-11011, Invitrogen) were used to detect CTC-TRPC5. CTC were visualized using a confocal laser scanning microscope (Leica TCS SP8, Wetzlar, Ger-many) and analyzed using the ImageJ software.

### Immunohistochemical Staining

CRC tissue slides were deparaffinized with xylene and rehydrated in a graded alcohol series. After incubation in 10% BSA, the slides were subsequently incubated with the primary antibody anti-TRPC5 (1:200) (PA5-77310, Invitrogen) overnight at 4°C in a humidified chamber, followed by secondary antibody (A0277, Beyotime Biotechnology, Nantong, Jiangsu Province, China) incubation at room temperature for 1 h. The immunostaining results were assessed by two pathologists. From each slide, five visual fields were selected. The scores were calculated using the German semi-quantitative scoring system (no staining = 0; weak staining = 1, moderate staining = 2, strong staining = 3) and the extent of stained cells (0% = 0, 1–24% = 1, 25–49% = 2, 50–74% = 3, 75–100% = 4) was determined. The final immunoreactive score of TRPC5 protein expression examination by immunohistochemical staining (IHC-TRPC5) was calculated by multiplying the intensity score with the score extent of stained cells, ranging from 0 (the lowest score) to 12 (the highest score).

### Statistical Analysis

The most appropriate cut-off values of CTC-TRPC5 and IHC-TRPC5 score were obtained by generating the receiver operating characteristic (ROC) curve. The disease free survival (DFS) is defined as the interval between the date of surgery and the date of recurrence or the time of the last follow-up. The relationship between the clinical-pathological factors of patients and DFS was determined using univariate analysis. Multivariate analysis was performed using the COX regression method in order to search for independent prognostic factors for DFS. The median DFS (mDFS) was calculated using the life table method. *p* < 0.05 was considered statistically significant.

## Results

### Patient Follow-Up Results

A total of 116 patients were selected for this study. Table 1 shows the specific clinical and pathological data. By the last follow-up, 76 patients had relapsed. A total of 102 patients received fluorouracil-based adjuvant chemotherapy for 2–6 months, and the other 14 patients received no adjuvant chemotherapy.

**TABLE 1:** Clinical and tumor characteristics of the 116 CRC patients.

	All Patients (*n* = 116)	
Characteristics	n	%
Age (years)	–	–
Mean	60.5	
SD	10.3	
≤ 60	54	46.6
> 60	62	53.4
Sex	–	
Male	61	52.6
Female	55	47.4
Tumor location	–	–
Colon	58	50.0
Rectal	58	50.0
T stage	–	–
T1+2	38	32.8
T3+4	78	67.2
N stage	–	–
N0	65	56.0
N1-3	51	44.0
TNM stage	–	–
I-II	65	56.0
III	51	44.0
Tumor differentiation	–	
Well or moderately	79	68.1
Poorly	37	31.9

### CTC-TRPC5 Level Was Associated With IHC-TRPC5 Level

**FIGURE 1:**
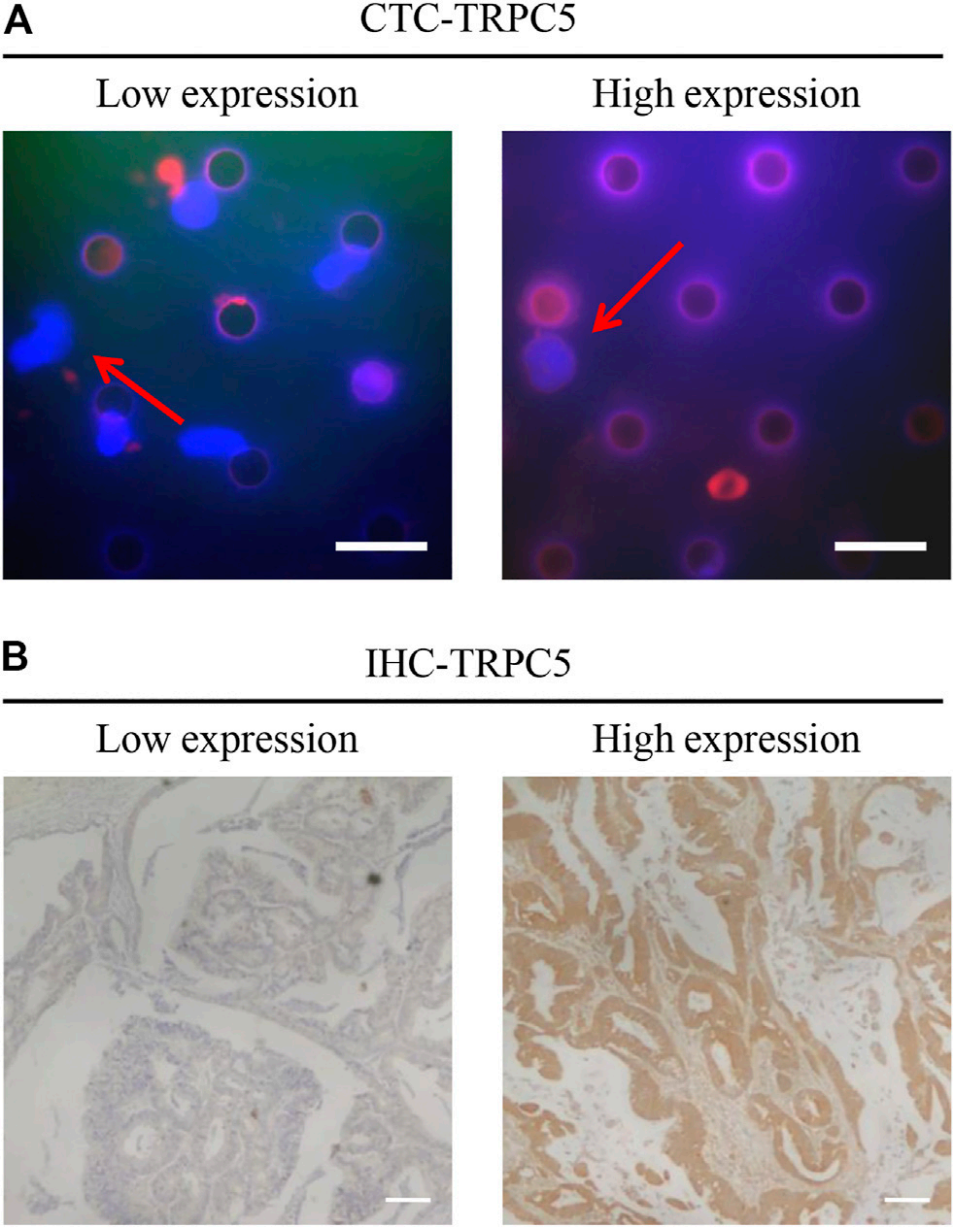
**(A)** Representative images of the immunohistochemical staining of CTC-TRPC5 expression (Scale bars, 20 µm). **(B)** Representative images of the immunohistochemical staining of TRPC5 expression in human CRC tissues (Scale bars, 300 µm).

**FIGURE 2:**
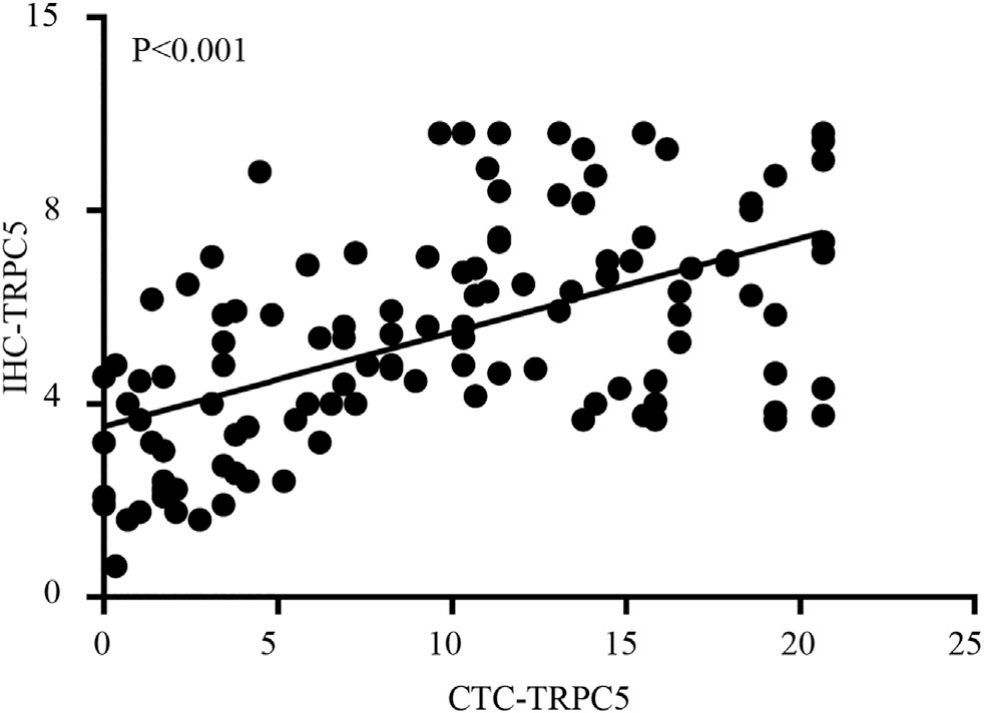
The Pearson correlation analysis significant relationship between CTC-TRPC5 and IHC-TRPC5 level.

TRPC5 protein expression by CTC was examined using immunofluorescence, while TRPC5 expression in the tumor tissue from the same patient was examined using immunohistochemistry. The representative images are shown in Figure 1 A and B. The Pearson correlation analysis showed significant relationship between CTC-TRPC5 and IHC-TRPC5 level (Figure 2).

### High CTC-TRPC5 level was significantly associated with a shorter DFS in radically resected CRC patients.

**FIGURE 3:**
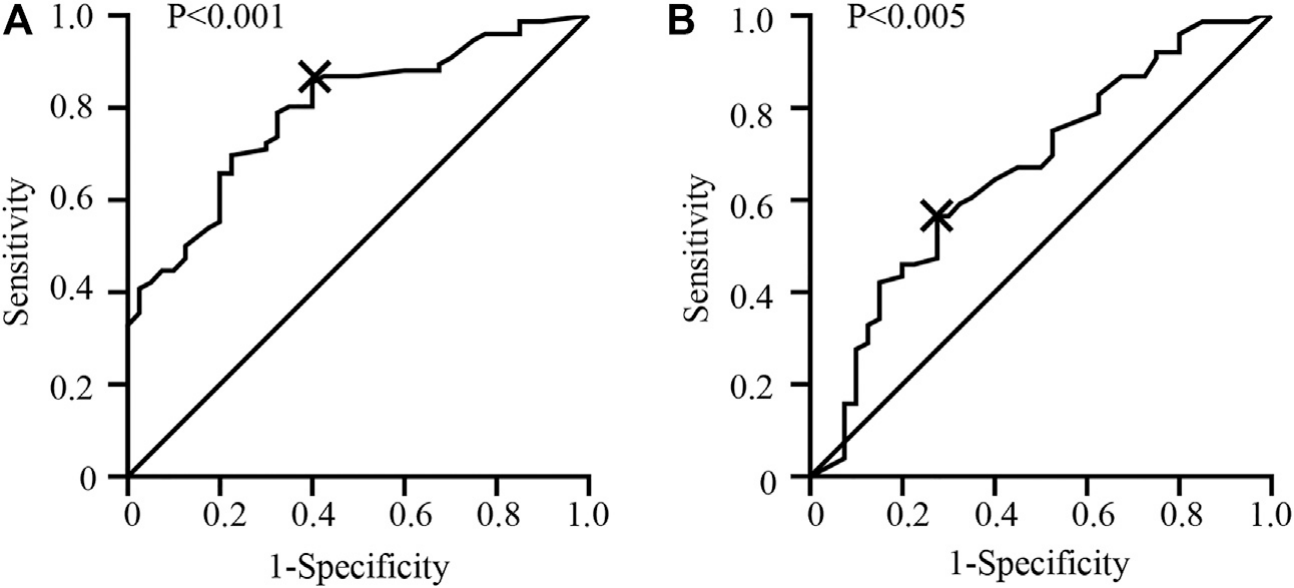
**(A)** ROC curve analysis identifying the appropriate cut-off value of CTC-TRPC5 score with 8.6 (AUC = 0.793; 95% CI = 0.712–0.875). **(B)** ROC curve analysis identifying the appropriate cut-off value of IHC-TRPC5 score with 8 (AUC = 0.664; 95% CI = 0.5581–0.7695).

**FIGURE 4:**
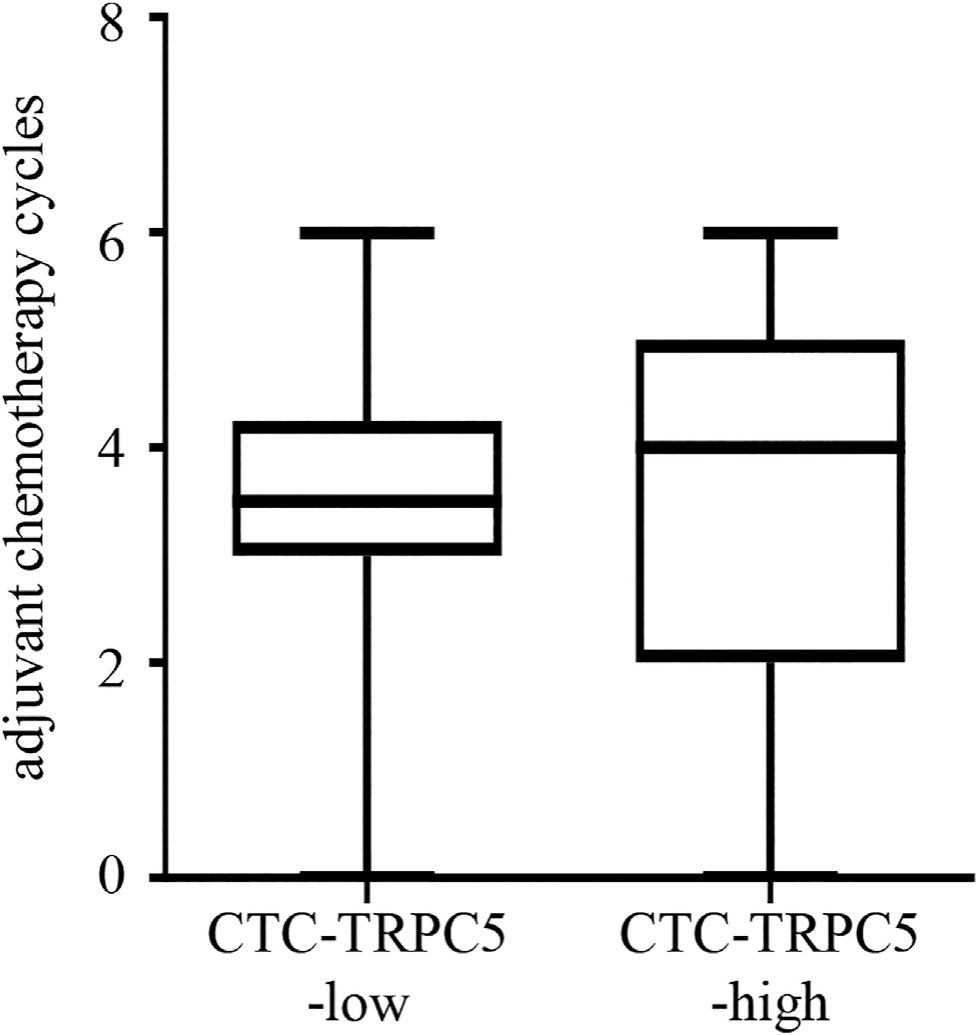
CTC-TRPC5 level was not significantly associated with the adjuvant chemotherapy cycles.

**FIGURE 5:**
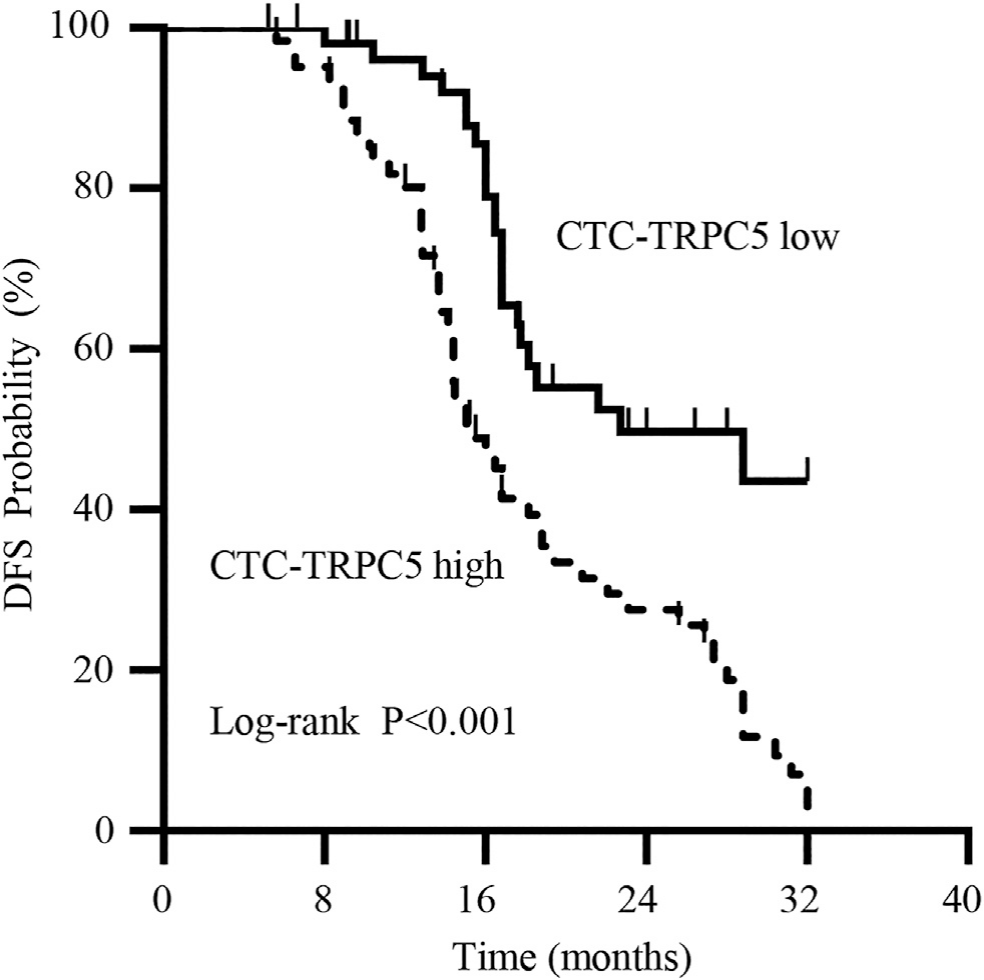
Kaplan-Meier survival curve showing a statistically longer median DFS in radical resected CRC patients with high CTC-TRPC5 expression than with those with low CTC-TRPC5 expression.

According to the ROC curve, the optimal cut-off value of CTC-TRPC5 for distinguishing high and low expression of CTC-TRPC5 was 8.6 (*p* < 0.001) (Figure 3A), and the optimal cut-off value of IHC-TRPC5 for distinguishing high and low expression was 8 (*p* < 0.005) (Figure 3B). According to the chi-square analysis, the CTC-TRPC5 level and IHC-TRPC5 level are both significantly related to the T stage and tumor differentiation, where CTC-TRPC5 levels and IHC-TRPC5 levels are higher in high T stage and poorly differentiated tumors (Table 2 and 3). In addition, CTC-TRPC5 level was not significantly associated with the adjuvant chemotherapy cycles (Figure 4), which were proved to reduce recurrence risk of radically resected CRC patients. The univariate analysis showed that high T staging, high N staging, poor differentiation, and initial high CTC-TRPC5 level were all significantly related to a shorter DFS. Furthermore, COX multivariate analysis revealed that high T staging, high N staging, poor differentiation, and initial high CTC-TRPC5 levels were all independent poor prognostic factors affecting DFS in CRC patients after radical surgery (Table 4). The mDFS of CRC patients with high and low CTC-TRPC5 levels was 17.1 and 22.0 months, respectively (*p* < 0.001) (Figure 5).

**TABLE 2:** Characteristics of CRC patients according to CTC-TRPC5 level.

Characteristics	CTC-TRPC5 expression		
	Low (n = 54)	High (n = 62)	*p* value
Age (years)	–	–	0.28
≤ 60	28	26	–
> 60	26	36	–
Sex	–	–	0.82
Male	29	32	–
Female	25	30	–
Tumor location	–	–	0.06
Colon	22	36	–
Rectal	32	26	–
T stage	–	–	0.01*
T1+2	24	14	–
T3+4	30	48	–
N stage	–	–	0.07
N0	35	30	–
N1-3	19	32	–
TNM stage	–	–	0.07
I-II	35	30	–
III	19	32	–
Tumor differentiation	–	–	< 0.01**
Well or moderately	45	34	–
Poorly	9	28	–

a *p* <0.05, **p <0.01.

**TABLE 3:** Characteristics of CRC patients according to IHC-TRPC5 level.

Characteristics	CTC-TRPC5 expression		
	Low (*n* = 61)	High (*n* = 55)	*p* value
Age (years)	–	–	0.35
≤ 60	29	25	–
> 60	32	30	–
Sex	–	–	1
Male	31	30	–
Female	30	25	–
Tumor location	–	–	0.70
Colon	29	29	–
Rectal	32	26	–
T stage	–	–	<0.01**
T1 + 2	24	14	–
T3 + 4	37	41	–
N stage	–	–	0.60
N0	34	31	–
N1-3	27	24	–
TNM stage	–	–	0.60
I-II	34	31	–
III	27	24	–
Tumor differentiation	–	–	0.01*
Well or moderately	44	35	–
Poorly	17	20	–

a *p* <0.05, **p <0.01.

**TABLE 4:** Univariate and multivariate analysis of factors associated with DFS.

Variables	Univariate analysis		Multivariate analysis	
	HR (95%CI)	*p* value	HR (95%CI)	*p* value
Age (years)	–	0.542	–	–
≤ 60	1	–	–	–
> 60	1.151 (0.732–1.810)	–	–	–
Sex	–	0.670	–	–
Male	1	–	–	–
Female	0.906 (0.575–1.427)	–	–	–
Tumor location	–	0.085	–	–
Colon	1	–	–	–
Rectal	0.671 (0.426–1.056)	–	–	–
T stage	–	0.004**	–	0.128
T1+2	1	–	1	–
T3+4	2.153 (1.277–3.628)	–	1.530 (0.884–2.646)	–
N stage	–	0.005**	–	0.038*
N0	1	–	1	–
N1-3	1.936 (1.225–3.059)	–	1.655 (1.027–2.665)	–
TNM stage	–	0.005**	–	0.038*
I-II	1	–	1	–
III	1.936 (1.225–3.059)	–	1.655 (1.027–2.665)	–
Tumor differentiation	–	< 0.001***	–	0.003**
Well/moderately	1	–	1	–
Poorly	2.609 (1.651–4.123)	–	2.078 (1.290–3.346)	–
CTC-TRPC5 level	–	< 0.001***	–	0.012*
High expression	1	–	1	–
Low expression	2.691 (1.647–4.398)	–	1.943 (1.156–3.265)	–

a *p* <0.05, **p <0.01, ***p <0.001.

## Discussion

CTC is a tumor cell that has separated from the primary or secondary tumor and has entered the circulatory system. Its advantages as a “liquid biopsy” include small trauma, reproducible detection, high specificity, and sensitivity, and can provide real-time information regarding the disease status of CRC patients. Information is useful for early screening, prognostic evaluation, and monitoring treatment response of CRC patients. Many studies have reported that CTC is significantly related to the prognosis of a variety of tumors. Goodman et al. discovered that breast cancer patients with positive CTC in the peripheral blood had a significantly worse prognosis than those with negative CTC(Goodman et al., 2018). In a previous study about CRC, it was also established that the presence of CTC in the peripheral circulation indicates a poor prognosis (Rahbari et al., 2010). Furthermore, CTC were found to be superior to traditional pathological staging as a prognostic factor (Iinuma et al., 2011;Bork et al., 2015). These findings might be the reasonable explanation of the seeming poor prognosis of the CRC patients enrolled in this study.

Many studies have shown that CTC exhibit a high degree of heterogeneity, with varying protein expression levels and localization (Keller and Pantel, 2019). Various subgroups of CTC may play different roles in the occurrence and development of CRC. These suggest that in addition to CTC number, this heterogeneous of CTC might have more potential clinical value (Fidler, 2003). Certain specific protein molecules expressed in CTC might represent specific CTC subgroup or exhibit unique biological behaviors, which have been studied in breast cancer (Hapach et al., 2021).

TRPC5 is a calcium ion channel expressed on the surface of cell membranes. Our previous studies have shown that TRPC5 has different biological functions in various tumors. For example, high expression of TRPC5 in CRC is related to drug resistance and dedifferentiation, which are linked to the poor prognosis of CRC patients (Wang et al., 2015; Chen et al., 2017). In this study, CRC patients who had undergone radical surgery were enrolled, with CTC in their peripheral blood collected and their TRPC5 protein levels measured. The findings revealed that CTC-TRPC5 levels were significantly related to the prognosis. The mDFS of CRC patients with high CTC-TRPC5 level was significantly lower than those with low or no TRPC5 expression, and multivariate analysis showed that high CTC-TRPC5 was an independent poor prognostic factor that affected the prognosis. These suggested that different CTC, which are a source of potential distant metastasis and recurrence, may have different abilities to cause a recurrence.

This study discovered that the TRPC5 level in tumor cells of CRC *in situ* was significantly correlated with the CTC-TRPC5 level in peripheral blood. This suggested that CTC-TRPC5 level could act as the real-time monitoring of TRPC5 expression of tumor cells *in situ*. Our previous study revealed that poorly differentiated CRC tissues expressed higher levels of TRPC5 in surgically removed colon cancer tissues (Chen et al., 2017). Combined with the findings of this study, CTC-TRPC5 expression in the peripheral blood of CRC patients after surgery was found to be significantly related to the differentiation of tumors. High levels of CTC-TRPC5 were more common in patients with poorly differentiated tumors. This suggested that poor differentiated CRC cells with high TRPC5 expression were more likely to be separated from solid tumor lesions and enter the blood circulation, where CTC are formed. This source of CTC may lead to a worse prognosis, and the mechanism of CTC formation with low TRPC5 levels remains unclear. Furthermore, the data analysis of this study showed that higher T staging is significantly related to higher CTC-TRPC5 levels, implying that another mechanism is involved, which needs further studies.

Previous and current researches on CTC mostly foused on their numbers. However, this study focuses on the heterogeneity of CTC populations and explores the clinical significance of specific CTC subgroups. CTC-positive CRC patients after radical resection were selected and examined TRPC5 expression of CTC. The results revealed that CTC with high TRPC5 expression indicated a worse prognosis. The number of cases included in this study is limited. Therefore, an increased number of samples, especially CTC-TRPC5 data in advanced CRC, as well as the inclusion of the overall survival data in future studies will provide more detailed clinical significance of the specific CTC subgroup in CRC. Taken together, our study found various TRPC5 expression level in different CTC and revealed the clinically significant CTC subgroup with high TRPC5 expression, providing a new indicator for clinical prognosis evaluation of radically resected CRC patients.

## Data Availability

The raw data supporting the conclusions of this article will be made available by the authors, without undue reservation.
